# Neglected Tropical Diseases: A Comprehensive Review

**DOI:** 10.7759/cureus.53933

**Published:** 2024-02-09

**Authors:** Jayashankar CA, Venkata Bharat Kumar P, Venkataramana Kandi, Girish N, Sanjana K, Divya Dharshini, Satya Vijaya Chandana Batchu, Prakash Bhanu

**Affiliations:** 1 Internal Medicine, Vydehi Institute of Medical Sciences and Research Center, Bangalore, IND; 2 Biochemistry, Vydehi Institute of Medical Sciences and Research Center, Bangalore, IND; 3 Clinical Microbiology, Prathima Institute of Medical Sciences, Karimnagar, IND; 4 Microbiology, Vydehi Institute of Medical Sciences and Research Center, Bangalore, IND; 5 Dermatology, Vydehi Institute of Medical Sciences and Research Center, Bangalore, IND; 6 General Medicine, Vydehi Institute of Medical Sciences and Research Center, Bangalore, IND

**Keywords:** who strategies, india’s ntd problem, global perspective, proverty-related neglected diseases, world health organization (who), controlling ntds, neglected tropical diseases (ntds)

## Abstract

Neglected tropical diseases (NTDs) are a group of diseases caused by diverse organisms, affecting millions of people in tropical and subtropical conditions. NTDs are more prevalent among people who live in poverty, without access to clean water, adequate sanitation, and quality health care. Most NTDs are chronic conditions and are potentially disablers than killers, leaving behind a trail of social consequences. Controlling NTDs has become complicated due to limited resources and are frequently ignored by global funding agencies. India experiences a significant burden of global NTDs. The paradox is that NTDs are preventable and treatable at an affordable cost. It then makes no sense as to why we co-exist with such diseases. The World Health Organization (WHO) has donned the leadership role of eliminating, eradicating, and controlling global NTDs. The WHO published a roadmap delineating a plan of action, which was being reviewed periodically. This led to substantive progress in tackling the NTDs. However, many challenges still exist to controlling and preventing NTDs. India has achieved significant progress towards NTD control and elimination by implementing the WHO strategies and action plans. This was evident by an increase in research and funding in this direction. The number of new drugs, vaccines, and investigative tools available and those in the pipeline is testimony to their efforts. Focusing singly on India’s NTD problem would substantially reduce the burden of poverty-related neglected diseases and could dramatically advance the global health agenda. This review highlights the problem of NTDs in the Indian and global perspective.

## Introduction and background

The world has been constantly ravaged by infectious diseases which have caused endemics (involve small geographical areas like a village, town, or a city and affect hundreds of people), epidemics (involve several geographical locations spread across a single country and affect thousands of people), and pandemics (involve several geographical areas spread across the globe involving several countries and affect millions of people). Some of these infectious diseases contributed to eliminating a chunk of the population. Additionally, these diseases, which have spread across countries involving different geographical regions, crippled the economies. The coronavirus disease 2019 (COVID-19) pandemic caused by the novel severe acute respiratory syndrome coronavirus-2 (SARS-CoV-2) is the latest example that contributed to severe morbidity and resulted in the death of millions of people.

Some infectious diseases caused by different microbial species belonging to bacteria, fungi, parasites, and viruses occur infrequently in different geographical regions, particularly affecting poverty-ridden populations. A few of these diseases are attributed to vectors like flies and larval forms of the flies and the involvement of animal hosts. Besides, some diseases are difficult to diagnose due to the unavailability of culture methods and diagnostic incapabilities. Furthermore, some infectious diseases are noticed in isolated cases and present sporadically. This results in healthcare workers having limited knowledge of the disease and its clinical features.

Hotez et al. introduced the term neglected tropical diseases (NTDs) to address such infectious diseases with the noble intention of propelling political momentum, funding, and research and development to help tackle the menace created by these diseases [1]. They are called neglected because these diseases fail to get attention on a global health agenda despite the current focus being universal health coverage.

There is no single consensus on the definition of NTDs. Different organizations, such as the World Health Organization (WHO), the Centre for Disease Control and Prevention (CDC), various non-government organizations (NGOs), and health administrators and experts, among others, have defined NTDs in their respective ways. WHO defined NTDs as a diverse group of transmissible diseases caused by a variety of pathogens, including viruses, bacteria, parasites, fungi, and toxins that affect more than 1.5 billion people in tropical and subtropical regions who live in poverty without access to clean water, adequate sanitation and quality health care [2].

There is also no consensus on which diseases be labeled as NTDs. Many diseases caused by the Ebola virus (EBV), Zika virus (ZKV), and coronaviruses, among others, are not on the WHO's list of NTDs. Several other agencies have their suggested list, different from the WHO's list of NTDs [3].

Much of the attention has been focused on intensifying resources for fighting the three most devastating diseases, like acquired immunodeficiency syndrome (AIDS) caused by the Human Immunodeficiency Virus (HIV), tuberculosis (TB), and malaria [1]. NTDs have limited resources and are almost ignored by global funding agencies. Reasons for this include poverty, geographical isolation, stigmatization, scarcity of data regarding local and global burden estimates, and insufficient political will and financial resources for their control. Further, there seems to be a lack of lobbies for the most vulnerable population groups who are most severely affected by these diseases. An increasing body of evidence indicates that NTDs threaten the health of poor people, as do other diseases like HIV/AIDS, TB, and malaria. Lack of timely access to treatment and care can be debilitating and stigmatizing, leading to lifelong disabilities and costing developing countries billions of dollars every year in managing NTDs.

## Review

Characteristics of NTDs

**Figure 1:**
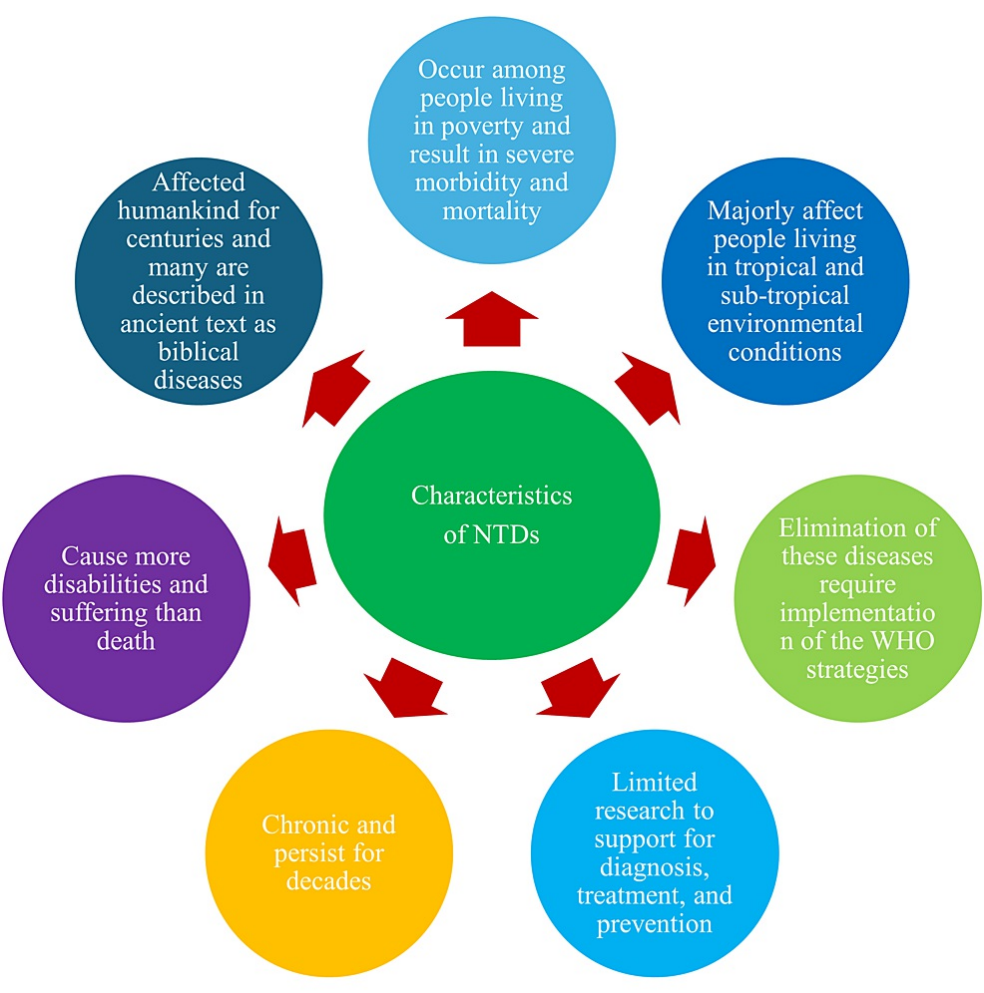
Characteristics of neglected tropical diseases (NTDs) Image credits: Venkataramana Kandi WHO: World Health Organization

The characteristics of diseases that enable them to be classified under NTDs include diseases that occur among people living in poverty and result in severe morbidity and mortality, majorly affect people living in tropical and sub-tropical environmental conditions, limited research to support for diagnosis, control, and prevention, and elimination of these diseases require the implementation of the WHO strategies. Other characteristic features of NTDs include diseases affecting humankind for centuries and described in ancient texts, also called biblical diseases that persist for decades, are chronic and cause more disabilities than the death of the affected persons [4-6]. The characteristics of NTDs can be depicted in Figure 1.

List of NTDs

**Figure 2:**
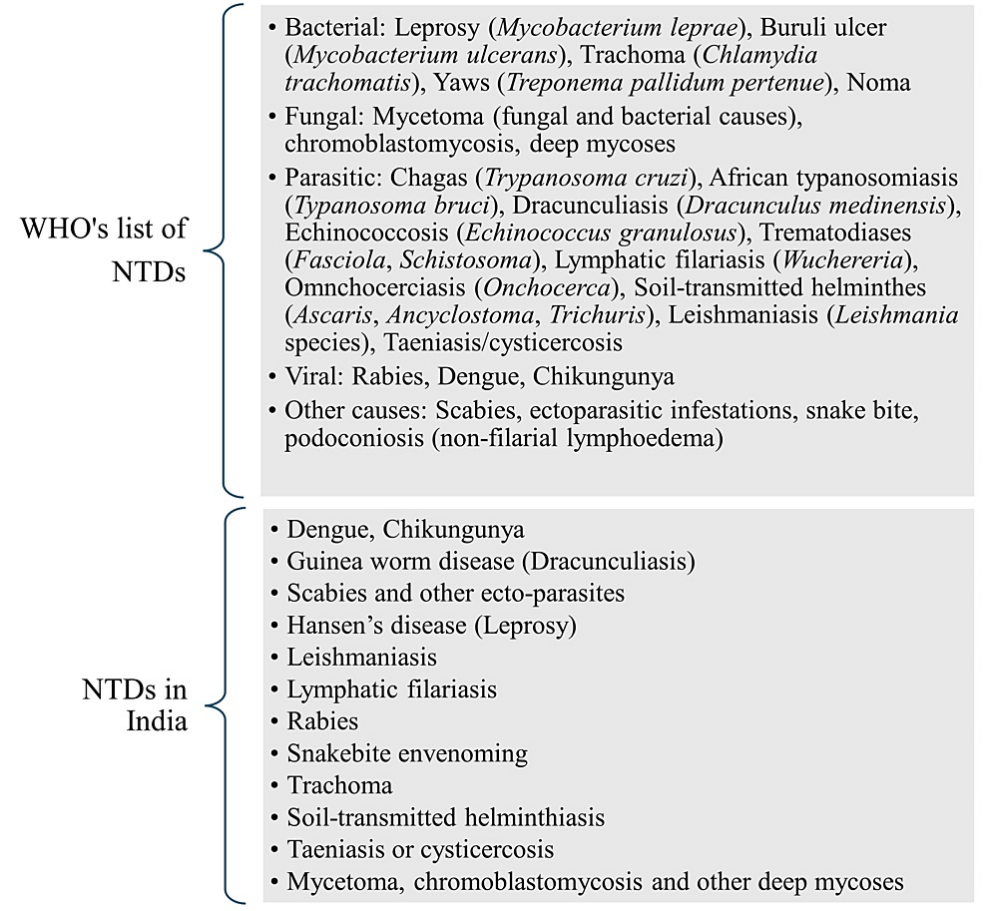
Neglected tropical diseases (NTDs) suggested by the World Health Organization (WHO) and those prevalent in India Image credits: Venkataramana Kandi

The list of NTDs initially consisted of 13 diseases, including seven helminthic parasites, three bacteria, and three protozoal infections. Later, the WHO expanded them to 17, which increased after 2016, by adding three more (2017: chromoblastomycosis and other deep mycoses, scabies, and snakebite envenoming) to make it 20 [2-4]. WHO amended the list in December 2023 by including noma, to increase the number of NTDs to 21 in total [7]. India suffers from the enormous burden of NTDs, wherein at least 12 different diseases are common in India. The list of NTDs suggested by the WHO and those that are prevalent in India are shown in Figure 2.

Consequences of NTDs

The consequences of NTD prevalence include their harmful effects on the course and outcome of pregnancy, disability, stigma, delayed physical and intellectual development during childhood, holding them out of school, reduced working productivity, forcing adults out of work, and inflicting burden on households with considerable costs to seek health care, Thus, the families, communities and ultimately the country could face social and economic difficulties [8].

In a global scenario, NTDs were estimated to affect close to two billion people, killing at least 200 thousand people each year from snakebite envenoming, rabies, and dengue alone, with a collective disability-adjusted life years (DALYs) burden that was equivalent to other diseases like HIV/AIDS, TB, and malaria which gained more attention [4].

Because of their economic excesses and strong political commitment, some developed nations have been able to overcome and keep these diseases in check, while many countries are still harboring these diseases and continue to suffer. Over time, the commitment to control NTDs has slowly waned due to economic, political, professional, and global reasons.

The Indian NTD scenario

India is the world's most populous nation, with 1.4 billion people accounting for 18% of the world’s population. India is the fifth largest economy in total gross domestic product (GDP) [9]. India experiences the world’s absolute burden of at least 11 main NTDs. Excluding NTDs mediated by unique hosts/insect vectors (schistosomiasis, onchocerciasis, human African trypanosomiasis, and Chagas disease), India leads the world in terms of the total number of cases for each of the primary NTDs, as defined by the WHO [10].

Among the NTDs prevalent worldwide, India accounts for a high burden of cases of visceral leishmaniasis, dengue, visual impairment from trachoma, leprosy, lymphatic filariasis, cysticercosis, and rabies. Additionally, up to one-quarter of the world’s ascariasis and hookworm cases are reported in India. Although no specific information about other diseases like amebiasis or giardiasis is provided, India may harbor a significant percentage of these cases that represent NTDs.

The World Health Organization (WHO) and NTDs

The London Declaration on NTDs was signed by stakeholders like the WHO, World Bank, pharmaceutical companies, and non-government organizations (NGOs) on January 30, 2012 [11]. A roadmap was proposed to eliminate, eradicate, and control at least 60% (10/17) of NTDs and improve the lives of over a billion people by 2020. Despite failing to achieve the desired results of the previous road map, the WHO, during the 73rd World Health Assembly, proposed a fresh road map to eliminate NTDs by 2030 and celebrate January 30 every year as the NTD day [12]. The road map builds on the achievements of the past ten years and the lessons learned to drive progress toward 2030 by identifying the main gaps and enforcing actions required to reach the targets by 2030 [13].

The targets and challenges of the 2030 road map

The overarching 2030 global targets are reducing the number of people requiring treatment for NTDs by 90%, DALYs related to NTD by 75%, eliminating at least one NTD from 100 countries, and eradicating two diseases (dracunculiasis and yaws) globally [2].

Many diseases that potentially fall into the category of NTDs are not included in established national policies and programs, probably due to inadequate funding. Research activities for NTDs have been stagnant for decades, sometimes becoming lesser every year. NTD research garners far less funding compared with other diseases like HIV/AIDS, TB, and malaria. Only 0.6% of development assistance for health is allocated to NTDs that affect about 1.5 billion people or roughly 20% of the world population [14].

Further, the activities under the NTD road map were affected by the COVID-19 pandemic. Activities with community-based interventions, such as preventive chemotherapy, diagnostic work-ups, active case-finding, etc., were severely affected by lockdown and movement restrictions. For example, in 2020, the number of people reached by mass treatment interventions dropped to 798 million from 1.207 billion in 2019 [2]. Although a resumption of activities has begun in 2021, the observed recovery is still partial and far from pre-COVID-19 levels. Delays in reporting, poor quality of data, and an inability to take timely corrective measures based on these data still exist.

A high index of suspicion is required among clinicians to include NTDs in their differential diagnosis. Clinicians should be trained to identify the classical clinical features and early complications like distant organ involvement and organ failures of some of these diseases.

A systematic review covering 25 years (1975-2000) revealed that virtually no new drugs were developed for NTDs [3]. Therefore, there is a need for research to invent efficacious, safe, easy-to-use, and affordable drugs. Further, simple easy to perform, reliable, and affordable laboratory investigations in place of complex, expensive, and less sensitive investigations like polymerase chain reaction (PCR), and nucleic acid sequence-based amplification (NASBA) which is beyond the reach of the people in the third world countries should be the focus of all clinicians. 

Sustainable development goals (SDGs)

The health-related SDGs include the implementation of the NTD agenda to reduce poverty and hunger, improve access to clean water, sanitation, and education, and enable decent work and economic growth, thereby reducing inequalities [15]. Consequently, control of NTDs appears to be essential for achieving these targets.

About 40 countries have eliminated at least one NTD. Dracunculiasis is nearing eradication, with 54 human cases reported in four countries in 2019. Nearly 16 countries have already eliminated lymphatic filariasis and trachoma as major public health problems, with more countries in the pipeline to achieve such targets. Further, some (four) American states have eliminated onchocerciasis.

The annual number of reported cases of human African trypanosomiasis has fallen from more than 7,000 in 2012 to fewer than 1,000 in 2018, eclipsing the original target of 2,000 cases by 2020. Similarly, the number of new leprosy cases reported globally has declined since 2010 at an average of 1% [13].

The Kigali declaration on NTDs

A global campaign aimed at 100% commitment was launched on World NTD Day in 2022, called the Kigali Declaration on NTDs to accomplish the target of eliminating NTDs. The campaign called for stakeholders to make bold financial and political commitments towards achieving the 2030 roadmap and SDG targets concerning NTDs [16].

**Figure 3:**
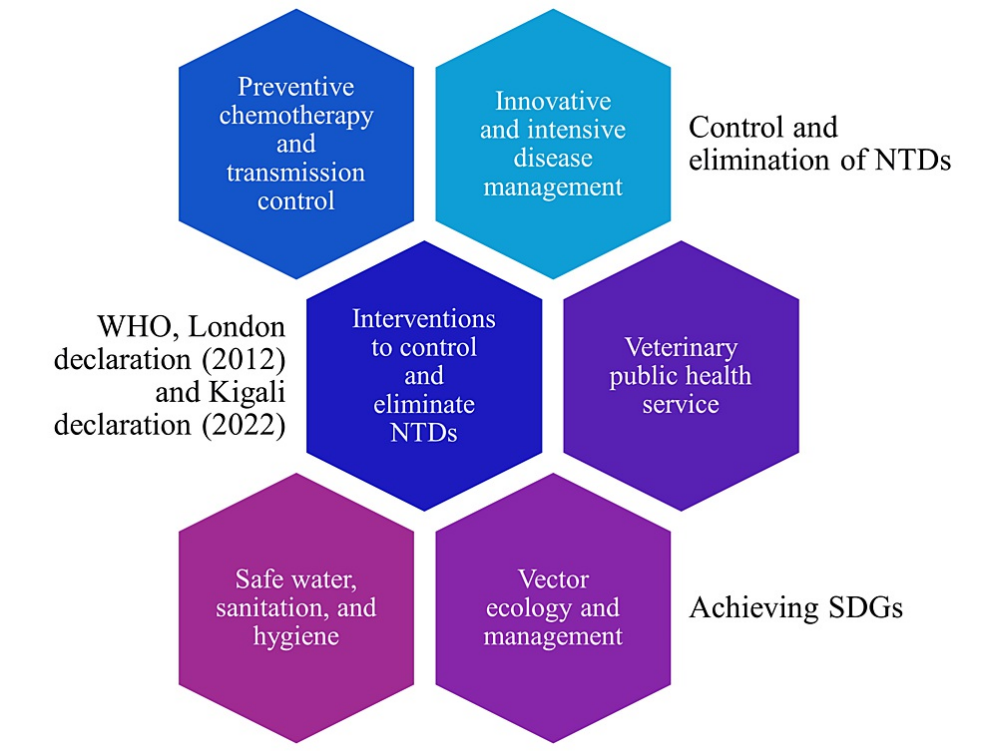
Interventions suggested by the WHO to achieve SDGs and control and eliminate NTDs Image credits: Venkataramana Kandi WHO: World Health Organization; NTDs: Neglected tropical diseases; SDGs: Sustainable development goals

Through the Kigali declaration, the WHO has suggested five interventions that could be implemented to achieve the 2030 goals. These include preventive chemotherapy and transmission control (PCT), innovative and intensive disease management (IDM), vector ecology and management, safe water, sanitation, and hygiene, and veterinary public health service (Figure 3).

The PCT program includes mass drug administration (MDA) and preventive treatment with drugs which resulted in NTD control [17,18]. Following this strategy and using MDA, six NTDs (dracunculiasis (guinea worm disease), lymphatic filariasis, onchocerciasis, schistosomiasis, trachoma, and soil-transmitted helminthic diseases caused by Ascaris, Ancyclostoma, and Trichuris) have been virtually controlled and eliminated in some parts of the world.

The WHO evolved programs were implemented by non-medical personnel like self-help groups, Accredited Social Health Activists (ASHAs), and schoolteachers, among others [19]. After updating with the latest drug regimens, the clinicians can pass on the material and information to the non-medical personnel to distribute among the general population [20].

The IDM allows health administrators to focus on the principle of early diagnosis and treatment of diseases. This enables the management of NTDs within the primary healthcare systems, thereby contributing to eliminating those diseases and preventing any future public health problems [4]. Vector management focuses on effective methods of targeting mosquitoes, flies, ticks, bugs, and other insect vectors that potentially transmit pathogens. One of the common principles employed is the effective use of pesticides [21]. Access to safe water, adequate sanitation, and hygiene are critical interventions to control and eliminate NTDs. According to the latest data, 40% of the world’s population lack handwashing facilities at home, 47% of schools lack handwashing facilities, and 16% of healthcare facilities have no functioning toilets or handwashing facilities. Globally, it can be observed that as the number of people move towards better living conditions, there has been a corresponding decrease in the number of NTDs [22]. The veterinary public health services recognize that people's health is connected to the health of animals and the environment in which people live. Many NTDs, such as rabies, are zoonotic diseases that are transmitted from animals to humans [23]. Therefore, it is suggested that human and animal health programs could be integrated to achieve NTDs that are potentially transmitted from humans to animals.

Results of the implementation of proposed strategies in India

The Indian perspective on overcoming the NTD menace has been going on for the last two decades, with targets being rewritten in 2015, 2018, and 2020. The latest is the new target set for 2030. Of around 12 NTDs prevalent in India, only dengue, rabies, snakebite, and leprosy are notifiable diseases. Therefore, it is hard to assess the real burden of NTDs in India. Additionally, no single organization or government agency monitors the NTDs. Yet, India has been able to eliminate certain infectious diseases like guinea worm, trachoma, and yaws by adapting the strategies laid down by the WHO [9]. However, India is still far from achieving the same results concerning other NTDs like leishmaniasis, filariasis, leprosy, snakebite, and soil-transmitted helminthic infections. This could dent the possibility for India to reach the target of NTD control and elimination by 2030.

According to the WHO estimates, in 2015, approximately 150 million Indian children received mass treatment for deworming intestinal helminth infections, and nearly 300 million people received treatment for lymphatic filariasis. Additionally, the implementation of a multi-drug therapy program covered leprosy, resulting in important public health gains. This level of MDA needs to continue to reach 100% of India's at-risk or infected population [10].

India’s contribution to global research and development of newer drugs (30% of global drugs), diagnostics (12% of global diagnostics), and vaccines (60% of global vaccines) is significant. India developed the world’s first vaccine, which is expected to play a significant role in global leprosy elimination [24].

India also contributes financially towards NTD research. The average funding by the Indian Council of Medical Research (ICMR) from 2008 to 2015 was about $26 million (£21m/€23m) per year, while the National Institutes of Health in the United States of America (USA) contributed over £1.3 billion per year [25]. The Indian pharmaceutical companies have filed fewer patents for new drugs and innovations, compared to those in the USA or China, which have been investing heavily in innovation [26].

India accounts for a significant portion of the world disease burden. Focusing on India’s NTD problem would substantially reduce the burden of poverty-related neglected diseases and could dramatically advance the global health agenda [10].

Other diseases/infections that warrant recognition as NTDs

Among other NTDs that are potentially being ignored are tungiasis and myiasis. Tungiasis is the infection/infestation of fleas belonging to Tunga penetrans. Fleas are obligate hematophagous ectoparasites that have the potential to infest humans and animals. Tungiasis is unfamiliar to physicians, and most cases remain undiagnosed. Therefore, the disease remains underreported [27]. Myiasis is another neglected disease that results from the infestation of fly larvae of the Diptera family. Most cases of myiasis are noted among people living in unhygienic environmental conditions that favor the growth of fly larvae. Different types of human myiasis infections include intestinal myiasis, which occurs after consuming food contaminated with fly larvae, and wound myiasis, wherein the fly larvae accidentally develop in the wound [28,29].

Rickettsial infections are vector-borne bacterial infections caused by different species of *Rickettsia. *These infections result in acute febrile illness. Untreated patients may suffer from serious complications. Most Rickettsial diseases occur among people living in poverty [30]. Scrub typhus is a bacterial infection caused by *Orientia tsutsugamushi*. The larval forms of trombiculid mites (*Leptotrombidium deliense*) known as chigger mites act as vectors in the transmission of scrub typhus, which is endemic to some geographical locations named as tsutsugamushi triangle and include regions of Southeast Asia, Indonesia, China, Japan, India, Pakistan, and northern Australia [31,32].

Strongyloidiasis, caused by *Strongyloides stercoralis, *is another NTD often ignored, majorly due to the self-limiting infections it causes in healthy individuals. However, *Strongyloides *infections among immunosuppressed populations can result in severe morbidity and mortality [33,34].

Despite its frequent reemergence since its discovery in 1976 in Zaire and the Democratic Republic of Congo, the EBV is another NTD that needs increased attention. EBV infection results in high mortality rates wherein the patients suffer from hemorrhagic complications. EBV outbreaks have been reported in countries (Guinea, Liberia, and Sierra Leone) other than those where the virus first emerged [35,36]. 

ZKV infection is another less-known tropical disease that must be included under the list of NTDs. ZKV is transmitted by mosquitoes belonging to *Aedes *species. Moreover, ZKV infections remain silent without any noticeable symptoms among infected populations. Additionally, ZKV infections were found to be associated with Guillain-Barré syndrome, complications in pregnancy, and fetal abnormalities [36].

A clinical condition named noma, a necrotizing bacterial disease that affects orofacial regions has recently been suggested as a potential NTD and was added to the WHO list of NTDs [7]. Despite satisfying all criteria set by the WHO, noma was ignored for a long time. Noma presents as necrotizing gingivitis with evidence of edema, gangrene, and scarring. This condition is more prevalent in people living in sub-Saharan Africa [37].

*Nocardia *are acid-fast bacteria associated with pulmonary infections similar to tuberculosis. Additionally, Nocardia species can cause mycetoma, which causes a common debilitating disease that presents as a chronic granulomatous condition in India and many other tropical and sub-tropical countries [38,39].

Leptospirosis is a zoonotic infection transmitted by rat urine and feces through water contamination. Infected humans develop jaundice and hemorrhagic complications of the eyes and kidneys. Due to the limitations associated with knowledge, prevalence, and diagnosis, leptospirosis remains underreported [40].

Tropical endomyocardial fibrosis is a severely debilitating and potentially life-threatening cardiovascular disease characterized by the fibrous transformation of the endomyocardium leading to cardiomyopathy [41].

Due to ignorance and the complexity of the disease, cryptococcosis has remained an NTD despite its vast prevalence in different geographical regions. *Cryptococcus *is a fungus present in the environment that has the potential to cause superficial to deep and systemic disease, especially among immunocompromized persons [42,43].

Carrion’s disease is caused by a bacterium named Bartonella *bacilliformis *and is transmitted to humans by the bite of sandfly *Lutzomyia *species. This disease is prevalent in some South American countries. This disease results in a hemolytic condition among humans, which is known commonly as Oroya fever, that has a mortality rate of up to 90% [44].

Melioidosis is a disease caused by the bacterium *Burkholderia pseudomallei*. Despite the endemic prevalence of this bacterium, melioidosis is neglected due to underdiagnosis, as reported by a recent case from Gujarat, India [45].

Melioidosis is otherwise named Whitmore's disease, which can be noticed in humans and animals and is majorly found in tropical and subtropical regions of the world with scanty reports from the United States of America (USA). Most humans acquire infection by being close to infected animals. *B. pseudomallei *causes disease among healthy people, but people with underlying diseases like thalassemia, cancer, diabetes, lung, liver, and kidney diseases, and immunocompromised individuals suffer from severe complications [46].

Hepatitis E virus (HEV) is a waterborne illness that results in jaundice. Despite the infection with HEV is self-limiting, untreated people may suffer from mortality up to 30%, especially among pregnant women [47].

Podoconiosis is non-filarial elephantiasis (tropical lymphedema) wherein patients suffer from lymphedema. It was noted among people who walk barefoot in soil contaminated with volcano dust. Podoconiosis results in acute dermatolymphangioadenitis, especially among poor people exposed to volcanic soils, owing to the inability to buy protective shoes [48].

Mansonellosis is a filarial disease caused by *Mansonella *(M) species, including *M*. *perstans*, *M*. *ozzardi*,* *and *M*. *streptocerca*. Despite being endemic to some geographical regions of Africa like Senegal and Zimbabwe, among others, the effect of this parasitic infection on human health is not adequately investigated. Additionally, this parasite affects people living in poverty [49].

Recommendations

NTDs disable and ruin society, country, and the world in all aspects of human lives, including the social and economic aspects, among others. The poor people, with little political voice, suffer most because of their low preference for public health decisions. A global response and a concerted strategy with a common approach to eliminate NTDs are warranted. This can be achieved under the leadership of the WHO [4]. Defining health system bottlenecks and addressing them at various levels (national, state, district, and even panchayat levels) are crucial for implementing successful control and preventive programs. Countries must show their political commitment and galvanize resources and opportunities in combating these diseases [50].

## Conclusions

NTDs are a group of infectious and non-infectious diseases that are prevalent throughout the world. Some NTDs are endemic to specific geographical regions and restricted to countries. Most NTDs are dominant in people belonging to low socioeconomic status. A vast majority of NTDs are neglected and do not get adequate attention from the health administrations. Additionally, many diseases that potentially fall into the category of NTDs remain underreported and ignored owing to a lack of definitive surveillance mechanisms and reporting systems. Since NTDs contribute to severe morbidity and result in DALYs, they could affect the workforce of individuals and affect the country’s economic growth. Timely diagnosis, initiating preventive measures like MDA, and educating people living in poverty about the role of nutrition, hygiene, and sanitation in preventing NTDs assume increased significance. Further, research activities are essential to developing specific therapeutics drugs and vaccines against the NTDs.
